# Characterization of Olive Fruit Damage Induced by Invasive *Halyomorpha halys*

**DOI:** 10.3390/insects14110848

**Published:** 2023-10-31

**Authors:** Elissa Daher, Elena Chierici, Stefania Urbani, Nicola Cinosi, Gabriele Rondoni, Maurizio Servili, Franco Famiani, Eric Conti

**Affiliations:** Department of Agricultural, Food and Environmental Sciences, University of Perugia, 06121 Perugia, Italy; elissa_93d@live.co.uk (E.D.); elenachierici9@gmail.com (E.C.); stefania.urbani99@gmail.com (S.U.); nicola.cinosi@studenti.unipg.it (N.C.); maurizio.servili@unipg.it (M.S.); franco.famiani@unipg.it (F.F.); eric.conti@unipg.it (E.C.)

**Keywords:** brown marmorated stink bug, feeding injury, *Olea europaea*, piercing–sucking, salivary sheath

## Abstract

**Simple Summary:**

The brown marmorated stink bug, *Halyomorpha halys* (Stål), is a highly polyphagous invasive species in Europe. This species can puncture fruits, causing fruit deformations and abscissions. Recently, *H. halys* was detected on olives in Greece and Italy. Injuries are being attributed to this pest by olive growers in northern and central Italy, but studies are necessary to confirm feeding and describe the damage. Here, we evaluated the effect of the artificial infestation of olive trees with different densities of *H. halys* on fruit drop and characterized the phenolic composition in injured vs. healthy fruits.

**Abstract:**

The brown marmorated stink bug, *Halyomorpha halys* (Stål), is an invasive species causing economic crop losses. This species was recently detected attacking olive fruits. The aim of this study was to characterize feeding damage. Olive samples were initially collected from a field where *H. halys* was reported to cause damage to olive fruits. Hence, we conducted a field trial on the Moraiolo variety using sleeve cages to test the effect of *H. halys* feeding pressure on olive fruit drop and evaluated the effect of feeding on fruit quality. We tested two densities of *H. halys* (two or eight adults/cage) at two different stages of olive development, pre- and post-pit hardening. High pressure of *H. halys* before pit hardening caused a significant fruit drop compared to the control. In addition, chemical analysis of damaged and infested fruits revealed higher levels of total phenols compared to healthy fruits. These findings indicate that feeding by *H. halys* induced a stress response in the plants that could translate in quality variations in the olive drupes.

## 1. Introduction

The risks imposed by invasive insects increase with climate change and global trading and have been estimated in a minimum of USD 70.0 billion of global losses per year [1,2]. Such losses are the consequence of direct feeding on marketable plant parts [3] and alteration in plant physiology and morphology [4]. In addition, unpredictable ecological effects in the new environment should be considered [5]. When introduced into a new habitat, if they find favorable climatic conditions, invasive species tend to thrive on the expense of indigenous species due to their superior abilities to escape control of natural enemies [6] and to win direct and indirect competition [6,7,8]. The brown marmorated stink bug (BMSB), *Halyomorpha halys* (Stål) (Hemiptera: Pentatomidae), is an invasive pest originating from Asia, after being accidentally introduced to the United States and Europe [9]. After more than a decade of its presence in Europe [10], major agricultural damages by *H. halys* were recorded in Italy [9,11], which required emergency measures in support of fruit and vegetable producers [12]. *Halyomorpha halys* is highly polyphagous with more than 300 hosts [13]; it is a pest of many fruit trees including apples, peaches, nectarines, pears, and grapes [9]. Haye et al. [14] listed olive (*Olea europaea* L.) as a crop potentially susceptible to *H. halys*. In fact, adults and juveniles *H. halys* were recently recorded on cultivated olives in Greece [15,16]. Concerns are increasing, especially after *H. halys* was reported to be damaging olives in the north of Italy [17]. Despite several sustainable pest control measures being available for olive pests, e.g., *Bactrocera oleae* Rossi [18,19], they are of limited efficacy against *H. halys* [20,21]. From its initial introduction point in the north of the country, *H. halys* has been detected in all Italian regions [11,22,23].

Similar to other phytophagous pentatomids, *H. halys* feeds on vegetative and reproductive plant parts by inserting the stylets in the tissue while ejecting two different kinds of saliva: readily solidifying saliva and watery saliva [24]. The solidifying saliva, also called sheath saliva, is responsible for the formation of a tubular lining, the so-called stylet sheath, salivary sheath, or salivary cone, while the watery saliva contains enzymes that help digest the plant tissue before ingestion [24,25]. After the extraction of the stylets, the salivary sheath, formed during feeding, remains attached to plant tissue. This type of feeding causes alterations to the surface and the inner part of the attacked fruit and could manifest as scars, discolorations, deformations, or occasional suberification of olive fruits attacked by *H. halys* [14,15]. In addition, the damage could be divided into two categories; the quantitative damage occurs early in the season, during rapid mitotic growth, leading to yield loss, while qualitative damage takes place during the fruit mesocarp development [9,26].

*Halyomorpha halys* feeding on fruit crops such as cherry and kiwi caused visible damages, fruit abscission, and reduced marketability. For instance, the damage on kiwi is characterized by green and white damage spots under the skin of kiwifruit while on cherry, scars and fruit drop were recorded [27,28]. On apples, *H. halys* feeding causes surface discolorations and visible stylet punctures with necrotic areas under the skin, where the same was seen on peaches with gummy areas on the external skin [29]. On olives, *H. halys* induced suberification in up to 87% of fruits [15]. Feeding by nymphs and adults can cause premature fruit drop [17]. However, to our knowledge, no studies have been conducted to compare the effect of early- or late-season attacks by different densities of *H. halys* on olive fruit drop and fruit chemistry. Many olive pests cause an abnormal increase in fruit drop, including the olive moth *Prays oleae* (Bern.) (Lepidoptera: Yponomeutidae) and the olive fruit fly *Bactrocera oleae* (Rossi) (Diptera: Tephritidae) [30,31], and *H. halys* could represent a potential additional cause of olive fruit losses [14,17].

Here, we first characterized the volatile profiles from olives (Casaliva and Leccino varieties) naturally attacked in the field. Then, we evaluated fruit drop and phenolic compositions in olives (Moraiolo variety) artificially exposed in the field to two densities of *H. halys* at pre- and post-pit hardening stages.

## 2. Materials and Methods

### 2.1. Insects

The colony of *H. halys* was started from individuals collected from the province of Modena and reared in controlled conditions (25 ± 1 °C, 60 ± 5% relative humidity, and 16 h:8 h light:dark). Individuals were maintained inside mesh cages (Kweekkooi 40 cm × 40 cm × 60 cm, Vermandel, Hulst, The Netherlands). Approximately 30–40 individuals were placed in each cage. A paper towel was placed on the bottom of the cage to provide hygienic support for food. All insect stages were provided with a diet consisting of organic tomato fruits, carrot roots, raw peanuts, sunflower seeds, and green bean pods [32,33]. An upside-down cotton-sealed jar on a Petri dish was used for water supply. The food was replaced every two days and glass jars for water supply were substituted on a weekly basis. For the experiment, reproductive individuals (approx. 3–4 weeks old) were starved 24 h (water provided) in smaller mesh cages (Kweekkooi 30 cm × 30 cm × 30 cm, Vermandel, Hulst, The Netherlands) prior to their transfer to the field.

### 2.2. Field Surveys in an Olive Orchard Naturally Attacked by Stink Bugs

Field surveys were conducted in an organic olive orchard (Casaliva and Leccino varieties) located in northern Italy (Raffa, Brescia Province, Italy; coordinates of the central area: 45.582036, 10.530811). This orchard was selected because of the presence of several adult and juvenile *H. halys* on the olives. Samplings were conducted on 5 October 2020. Random olive collection was conducted on both varieties (approximatively 2 kg fruits per variety). Fruits were transferred to the laboratory and observed under a stereomicroscope. Olives were then divided in three groups according to the level of stink bug attack, i.e., “healthy” (no visible damage), “damaged” (only salivary cones were present), or “highly damaged” (with skin depressions, discolorations, and salivary cones). Olive samples were subjected to chemical analyses of the phenolic composition for assessment of the oil quality or molecular analyses for the diagnosis of the putative *H. halys* attack (see Section 2.4).

### 2.3. Field Assessment of H. halys Feeding Damage on Olive Fruits

In order to determine the effect of *H. halys* feeding on olive fruit drop, we conducted a field experiment in one olive orchard managed under integrated pest management systerm (Moraiolo variety) located in central Italy (Umbertide, Perugia Province, Italy; coordinates of the central area: 43.284183, 12.395923). The trial was carried out in two different stages of the fruit development: pre-pit hardening (from 28 July 2021) and post-pit hardening (from 29 September 2021). Due to the particular climatic conditions of the area, the pre-pit hardening was slightly delayed compared to other areas of central Italy, where it usually occurs about two weeks earlier. One branch bearing completely healthy olive fruits, i.e., without any visible injury or pest infestation, was selected. Each selected branch carried an average of 50 or 62 total fruits per branch for the pre- and the post-pit hardening trial, respectively. Two densities of *H. halys* adults, two (“HH2”) and eight (“HH8”) adults per olive branch, with a 1:1 sex ratio, were tested and compared to the control (no insects; “CNT”). Sleeve cages (75 cm height, 50 cm width) were used for the treatments and control. Cages were made of white tissue net (2 mm holes) to minimize the formation of a microclimate around the isolated branch and to permit aeration, but to also avoid any movement of olive pests to and from the branch.

Starved *H. halys* adults were transported to the field and were introduced in the cages. Feeding on olives was allowed for 48 h, then the insects were removed and returned to the laboratory. Mesh cages were kept until the end of the trial, i.e., the harvest day (8 November 2021) [26,27]. The cages were checked every two to three weeks and dropped fruits were collected, counted, and analyzed under a stereomicroscope for the presence of the salivary sheaths. When present, the drupes were dissected to investigate the extent of the damage in the mesocarp (pulp) and the endocarp (the pit). Close-ups of the salivary cones and dissected drupes were taken using a digital camera (Olympus) connected to a stereomicroscope.

Cages were randomly distributed among plants and treatments were randomly distributed among cages. For the pre-pit hardening feeding experiment, the number of replicates (cages) per each density were uneven due to unexpected *H. halys* mortality during the experiment and was five for CNT, four for HH2, and three for HH8. For the post-pit hardening test, all treatments had six replicates. Harvested olives, excluding dropped fruits, were subjected to chemical analyses of the phenolic composition.

### 2.4. Chemical and Molecular Investigation of H. halys Feeding Damage

Chemical investigations (phenolic composition) were conducted on olives collected in 2020 (Casaliva and Leccino varieties, see Section 2.2; Table S1) and in 2021 (Moraiolo variety, see Section 2.3). Phenols were extracted from the olive pulp according to the procedure described by [34] and modified as follows: 2 g olive pulp was homogenized with 50 mL of the methanol/water (80/20 (*v*/*v*) solution containing 20 mg/L of butylated hydroxytoluene (BHT) and homogenized using an Ultra-Turrax T50 (IKA, Werke, Staufen, Germany) for 1 min at 7000 rpm, then centrifuged at 9000 rpm for 10 min and the supernatant was recovered. The extraction was repeated twice and the extract was concentrated using a rotary evaporator (Buchi Rotavapor R-210, Buchi Italia srl, Cornaredo, Italy) until it reached a final volume of 20 mL. Phenols present in the aqueous extract were recovered via solid-phase extraction (SPE). For this purpose, a Bond Elut Jr-C18 and 1 g cartridges (Agilent Technologies, Santa Clara, CA, USA) previously activated with 10 mL of methanol and 10 mL of water was used. It was loaded with 2 mL of aqueous extract and the elution was performed with 50 mL of methanol; the eluate was evaporated to dryness using a rotary evaporator. The phenolic extract was solubilized in 1 mL of the methanol/water (50:50 *v*/*v*) solution, filtered using a polyvinylidene fluoride (PVDF) syringe filter (0.2 µm) (Agilent Technologies, Santa Clara, CA, USA), and injected into HPLC. The analysis of phenols of the extract was carried out according to Selvaggini et al. (2014) by using an HPLC Agilent Technologies system 1100 series (Agilent Technologies, Santa Clara, CA, USA) composed of a vacuum degasser, a quaternary pump, an autosampler, a thermostated column compartment, a diode array detector (DAD), and a fluorescence detector (FLD) and by also employing a C18 column Spherisorb ODS-1 (250 mm × 4.6 mm, 5 μm particle size) (Waters S.p.A., Milan, Italy). The phenolic compounds from olive pulp were detected by using a DAD set at a wavelength of 280 nm. The quantitation of phenols was determined by means of single calibration curves for each compound and the results were expressed as mg/g.

Five attacked olives for each of Casaliva and Leccino cultivars that were collected in 2020 were analyzed with PCR to confirm whether the feeding punctures were produced by *H. halys*. The positive control consisted in five green beans (*Phaseoulus vulgaris* L.), which were exposed for 12 h to *H. halys* adult feeding in a laboratory-controlled environment (25 ± 1 °C, 60 ± 5% relative humidity, 16 h:8 h light/dark). Fruits were first individually washed with a 0.1% NaClO solution for 1 min, then rinsed three times with distilled water. The NaClO treatment allows for the removal of degradation of contaminating DNA from the surface without precluding the successful purification of the nucleic acids from the sample [35]. A portion of olive pulp (5 mm diameter × 5 mm length) containing a salivary cone was then collected using a small brass corer. Total DNA was purified from the olive pulp using GenElute Genomic DNA kit (Sigma-Aldrich, Milan, Italy), adopting the manufacturer’s protocol. PCR amplification was conducted using procedure and species-specific primers for *H. halys* (*H. halys*-For and *H. halys*-Rev [36]).

### 2.5. Statistical Analysis

For 2021 field trials, the effect of *H. halys* density on the percentage of dropped fruits was evaluated by means of generalized linear models for the percentage data (GLMs, Beta distribution), followed by a multiple comparisons procedure and Holm correction (significance level, alpha = 0.05). Fruit weight and phenolic abundance in *H. halys*-infested drupes were evaluated separately for the pre-pit and post-pit fruit hardening stage by means of linear models (LMs). The data were analyzed in R using packages “betareg” [37] and “emmeans” [38].

## 3. Results

### 3.1. Field Surveys in Olive Orchards Naturally Attacked by Stink Bugs

Overall, the percentage of attacked fruits revealed via morphological analysis of the collected olives was 3% for Casaliva and 16% for Leccino. The composition of different phenols was variable in Casaliva and Leccino healthy and damaged olives (results in Table S1). Molecular diagnostics confirmed punctures on olive fruits by *H. halys* in 60% of the samples (Casaliva) and in 40% of the samples (Leccino). All green bean samples used as the control were positive to *H. halys*.

### 3.2. Field Assessment of H. halys Feeding Damage on Olive Fruits

**Figure 1:**
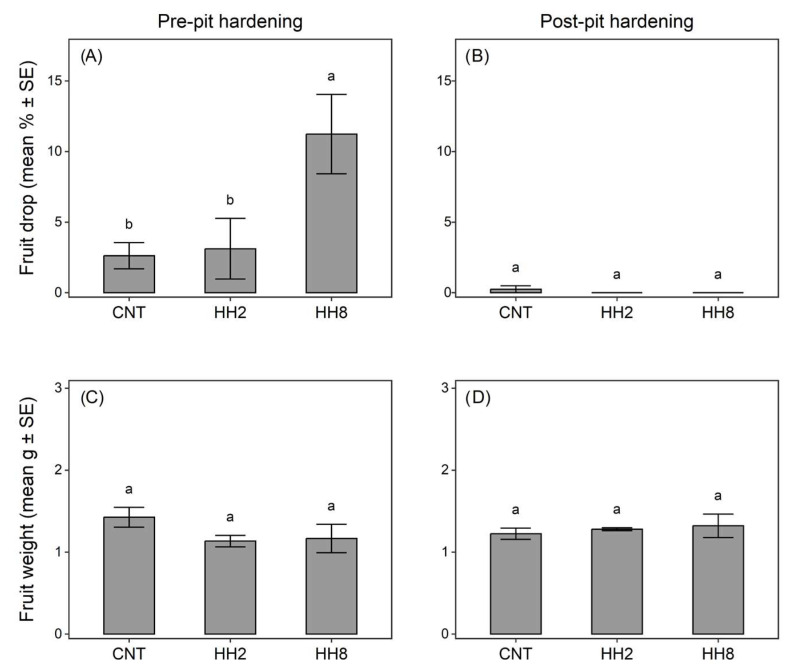
Effect of *Halyomorpha halys* density on olive fruit drop (**A**,**B**) and fruit weight (**C**,**D**) during pre-pit (**A**,**C**) or post-pit (**B**,**D**) fruit hardening. Tree branches were enveloped in sleeve cages containing 0 (CNT), 2 (HH2), or 8 (HH8) *H. halys* adults for 48 h. Mean values followed by different letters are significantly different for *p* ≤ 0.05 according to generalized linear models and multiple comparisons procedure.

In Moraiolo, the presence of the high density of *H. halys* (HH8) during the pre-pit hardening stage induced higher fruit drop compared to the low density (HH2) or control (results of GLM followed by a multiple comparisons procedure are reported in Figure 1). Conversely, fruit drop in HH2 did not differ from the control (Figure 1A). When olives were exposed to *H. halys* attack during the post-pit hardening stage, almost no fruit drop was recorded (Figure 1B). Regardless of the period of attack, fruit weight did not differ among treatments (Figure 1C,D).

**Figure 2:**
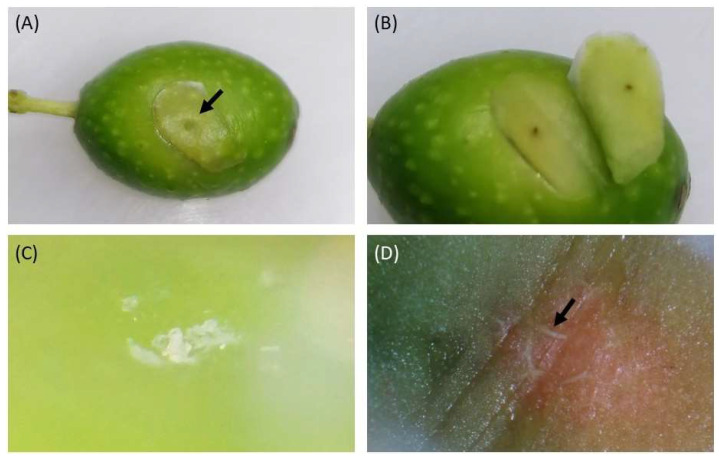
Close-ups of feeding puncture by *Halyomorpha halys* on olive fruits, with details of the visible feeding puncture (arrow) (**A**,**B**), of the feeding tube (salivary sheath) on fruit surface (**C**), and of the salivary sheath tube (arrow) penetrating the pulp and reaching the endocarp (**D**).

In both treatments (HH2 and HH8), the damage caused by *H. halys* was only detectable under a stereomicroscope, with some fruits exhibiting salivary cones and salivary sheaths as illustrated in Figure 2.

### 3.3. Chemical and Molecular Investigation of H. halys Feeding Damage

As per Moraiolo variety (2021 field experiment), some phenols as well as the sum of all phenolic fractions were different between olive fruits exposed to high density *H. halys* adults compared to the control, both in the pre- and post-pit hardening stages (Table 1 and Table 2). Attacks during the pre-pit hardening resulted in higher oleuropein in HH8, compared to HH2 and CNT. Verbascoside was higher in both HH8 and HH2 compared to CNT. The sum of the phenolic fractions was higher in HH8, followed by HH2 and then by CNT. Attacks during the post-hardening resulted in higher oleuropein in HH8 compared to HH2 and CNT. Verbascoside and the total phenolic extractions were higher in HH8 and HH2 compared to CNT.

## 4. Discussion

Here, we provided a comprehensive assessment of the effect of feeding by *H. halys* on olives. Olive fruits of Moraiolo were susceptible to fruit drop during the pre-pit hardening stage but not during post-pit hardening stage. The fact that during an early stage of development the feeding injury can cause fruit to abort and eventually drop was found for many plant species, such as apple, peach, and cherry attacked by *H. halys* [26,28]. Lately, this was observed on olives where feeding by adults *H. halys* was responsible for more than 66% of olive dropping [17]. The digestive enzymes discharged via saliva [24], with the absence of the endocarp lignification that is supposed to protect the seed [39], could easily harm the seed and lead to abortion. On cherries, *H. halys* feeding before pit hardening caused 100% abscission in fruits [28]. *Nezara viridula* L. was found to cause pre-mature dropping of citrus fruits via the same feeding mechanism [40]. In Australia, the stink bug was also associated with high damages to olive fruits [41].

The early stages of fruit development, i.e., flowering and early fruit growth, could also be critical since they coincide with the end of dormancy of *H. halys* (from April onward) [42]. Although no information is available regarding olive tree, any supposed feeding activity by stink bugs on flowers can cause fruit abortion and yield losses [26,42]. Our study did not cover the flowering stage and early fruiting bodies and further investigation is therefore needed. On the other hand, the olive variety Moraiolo has a relatively high detachment force with respect to other common varieties in central Italy such as Frantoio and Leccino [43]. In this context, the absence of fruit drop during post-pit hardening in Moraiolo should not be generalized to all olive cultivars and further studies are needed to draw conclusions. This study also concerned adult feeding, while nymphs were also shown to attack fruits, including olives [17,27]. Nonetheless, the results obtained contribute to the determination of olive vulnerable development stages, providing useful inputs for pest management decisions against *H. halys*.

The density of *H. halys* adults used in the experiment was also a determining factor along with the plant development stage. We observed that only high density caused significant fruit drop, where even a lower density of adults could cause a relevant percentage of dropping fruits [17]. The correlation between density and damage is also demonstrated in other studies conducted on different fruits. For instance, damage by *H. halys* on kiwi were higher with four adults per cage, rather than two [27]. The same correlation between density of *H. halys* adults and damage was also observed in blueberries [44]. The findings of these studies could be alarming because in the case of an outbreak of *H. halys*, accompanied with a high pest population density, a significant olive drop is to be expected.

The feeding injury was not easily detectable on Moraiolo fruits artificially exposed to *H. halys* because salivary cones were not always detectable under a stereomicroscope. However, the absence of salivary cones from the surface of fruits does not indicate the absence of the attack. In fact, sometimes deposits of the sheath material could be undetectable and sometimes insignificant to non-existent, despite the penetration of the stylets in the plant tissue [25]. Dissections of fallen fruits confirmed the ability of the stink bug to penetrate the mesocarp reaching the pit, enforcing the hypothesis of fruit abortion. On the contrary, visible punctures were observed on the collected fruits from the Leccino and Casaliva varieties. The different damage could be due to many elements: difference in the susceptibility between cultivars, the intensity of the attack, and the stage of development of olives when the infestation occurred. The Leccino and Casaliva fruits were collected late in the season (October 2020), making us assume that a long-term exposure to *H. halys* attacks until late in the season could have been the cause of the severe fruit deformation observed [28].

From the chemical point of view, fruits exposed to *H. halys* adults both in the pre-pit and in the post-pit hardening stage reported higher levels of phenolic compounds compared to the controls. Particularly, in pre-pit hardening stage, fruits exposed to the higher density of *H. halys* showed a greater amount of phenolic compounds compared to both the other treatment and the control. The increases in phenolic content in fruits during their development is also a defense mechanism adopted by the plant against herbivore attacks, and could indicate that the plant is under stress [45,46]. This might explain the amount of phenols in fruits highly damaged by *H. halys* and especially those in the pre-pit hardening stage. Plants have already been shown to respond to *H. halys* attacks via the activation of direct and indirect defense mechanisms (e.g., [24,33,47,48,49,50]). The defense mechanism costs the plants a reallocation of resources and could have a negative impact on plant physiology, growth, and productivity [46,51]. Alteration of the phenolic composition due to *H. halys* feeding was also reported for Istrska belica and Pendolino olive cultivars [52]. The amount of phenols, which includes six different families of compounds, is solidly related to the olive cultivar and fruit stage maturation [53,54,55,56]. One of these fractions, oleuropein, is the main phenolic compound of olive fruits, and its content tends to rise during the growth phase and decreases rapidly during the physiological development of the fruit [55,57,58]. Oleuropein belongs to the secoiridoid compound family, is present throughout the olive tree, and it is responsible for the bitter taste of unripe olives [59]. In addition to oleuropein, other phenolic compounds could be found, including verbascoside. Verbascoside is a heterosidic ester of caffeic acid and hydroxytyrosol and is found in a wide range of species within the Oleaceae family [52,59]. The increase in verbascoside in infested olives at the post-pit hardening stage is in line with its significant role in induced resistance. When *Agrilus planipennis* Fairmaire larvae were fed with a diet added with verbascoside, their survival and growth decreased in a dose-dependent way [60]. In another study, the compound demonstrated to impair insect growth and mortality of *Spodoptera frugiperda* (Smith) and *Drosophila melanogaster* Meigen [61]. Nevertheless, many factors could determine the phenol content such as the cultivar and the fruit size [53]. Regarding olives, the progressive development of oleuropein and verbascoside, along with their biochemical interrelation, could imply the presence of a metabolic connection between them. However, the exact causes of this relationship remain to be determined [53].

In conclusion, this study provides an assessment of the effect of *H. halys* feeding on olives. In the pre-pit hardening stage, *H. halys* feeding is critical. At the higher density of *H. halys* tested, damage could be both quantitative and qualitative, as demonstrated by the remarkable fruit drop and morphology of olive fruits and the alteration in oleuropein and verbascoside levels. Considering that hydrophilic phenols are essential for a good quality of virgin olive oil [62], further evaluation of the effect of *H. halys* attacks on oil quality should be carefully evaluated.

## Figures and Tables

**Table 1:** Phenolic composition (mg/g fresh fruits) of olives treated in pre-pit hardening stage. Means within a row followed by different letters are significantly different for *p* ≤ 0.05 according to linear model and multiple comparisons procedure.

	CNT	HH2	HH8
3,4-DHPEA	0.11 ± 0.019 b	0.08 ± 0.010 b	0.19 ± 0.020 a
*p*-HPEA	0.06 ± 0.011	0.06 ± 0.017	0.05 ± 0.013
Demethyloleuropein	n.d.	n.d.	n.d.
Verbascoside	1.70 ± 0.099 b	3.55 ± 0.555 a	4.54 ± 0.615 a
3,4-DHPEA-EDA	5.32 ± 0.478	6.53 ± 0.802	4.42 ± 0.767
Oleuropein	8.09 ± 0.355 b	7.2 ± 0.510 b	12.95 ± 2.188 a
*P*-HPEA-EDA	n.d.	n.d.	n.d.
Ligustroside	1.86 ± 0.177	2.35 ± 0.147	1.93 ± 0.145
Rutin	1.02 ± 0.045	1.38 ± 0.161	1.25 ± 0.055
(+)-1-Acetoxypinoresinol	n.d.	n.d.	n.d.
(+)-Pinoresinol	n.d.	n.d.	n.d.
Sum of the phenolic fractions	18.16 ± 0.123 c	21.14 ± 0.631 b	25.34 ± 1.55 a

**Table 2:** Phenolic composition (mg/g fresh fruits) of olives treated in post-pit hardening stage. Means within a row followed by different letters are significantly different according to linear model and multiple comparisons procedure.

	CNT	HH2	HH8
3,4-DHPEA	0.13 ± 0.010	0.11 ± 0.005	0.14 ± 0.006
*p*-HPEA	0.07 ± 0.010	0.05 ± 0.016	0.04 ± 0.003
Demethyloleuropein	n.d.	n.d.	n.d.
Verbascoside	0.95 ± 0.036 b	2.37 ± 0.166 a	2.17 ± 0.198 a
3,4-DHPEA-EDA	4.67 ± 0.789	5.64 ± 0.366	5.15 ± 0.383
Oleuropein	5.99 ± 0.509 b	6.44 ± 0.378 b	10.10 ± 1.275 a
*P*-HPEA-EDA	n.d.	n.d.	n.d.
Ligustroside	1.43 ± 0.197	1.91 ± 0.170	1.63 ± 0.087
Rutin	0.98 ± 0.115	1.15 ± 0.126	0.99 ± 0.052
(+)-1-Acetoxypinoresinol	n.d.	n.d.	n.d.
(+)-Pinoresinol	n.d.	n.d.	n.d.
Sum of the phenolic fractions	14.21 ± 0.796 b	17.68 ± 0.713 a	20.22 ± 1.090 a

## Data Availability

The data presented in this study are available on request from the corresponding author.

## Supplementary Materials

The following supporting information can be downloaded at: https://www.mdpi.com/article/10.3390/insects14110848/s1, Table S1: Phenolic composition (mg/g of fresh weight) of healthy and damaged olive fruits of cultivars Casaliva and Leccino (results are the mean of two independent extractions, each analyzed in duplicate).

## References

[1] Bradshaw C.J.A., Leroy B., Bellard C., Roiz D., Albert C., Fournier A., Barbet-Massin M., Salles J.M., Simard F., Courchamp F. (2016). Massive yet grossly underestimated global costs of invasive insects. Nat. Commun..

[2] Skendžić S., Zovko M., Živković I.P., Lešić V., Lemić D. (2021). Effect of climate change on introduced and native agricultural invasive insect pests in Europe. Insects.

[3] Battisti A., Benvegnu I., Colombari F., Haack R.A. (2014). Invasion by the chestnut gall wasp in Italy causes significant yield loss in *Castanea sativa* nut production. Agric. For. Entomol..

[4] Reale L., Tedeschini E., Rondoni G., Ricci C., Bin F., Frenguelli G., Ferranti F. (2016). Histological investigation on gall development induced by a worldwide invasive pest, *Dryocosmus kuriphilus*, on *Castanea sativa*. Plant Biosyst..

[5] Snyder W.E., Evans E.W. (2006). Ecological Effects of Invasive Arthropod Generalist Predators. Annu. Rev. Ecol. Evol. Syst..

[6] Oliveira C.M., Auad A.M., Mendes S.M., Frizzas M.R. (2013). Economic impact of exotic insect pests in Brazilian agriculture. J. Appl. Entomol..

[7] Rondoni G., Onofri A., Ricci C. (2012). Differential susceptibility in a specialised aphidophagous ladybird, *Platynaspis luteorubra* (Coleoptera: Coccinellidae), facing intraguild predation by exotic and native generalist predators. Biocontrol Sci. Technol..

[8] Kenis M., Auger-Rozenberg M.A., Roques A., Timms L., Péré C., Cock M.J.W., Settele J., Augustin S., Lopez-Vaamonde C. (2009). Ecological effects of invasive alien insects. Biol. Invasions.

[9] Leskey T.C., Nielsen A.L. (2018). Impact of the Invasive Brown Marmorated Stink Bug in North America and Europe: History, Biology, Ecology, and Management. Annu. Rev. Entomol..

[10] Wermelinger B., Wyniger D., Forster B. (2008). First records of an invasive bug in Europe: *Halyomorpha halys* Stål (Heteroptera: Pentatomidae), a new pest on woody ornamentals and fruit trees?. Mitteilungen-Schweiz. Entomol. Ges..

[11] Bariselli M., Bugiani R., Maistrello L. (2016). Distribution and damage caused by *Halyomorpha halys* in Italy. EPPO Bull..

[12] (2020). Commission Implementing Regulation (EU) 2020/465 of 30 March 2020. https://eur-lex.europa.eu/legal-content/EN/TXT/PDF/?uri=CELEX:32020R0465&from=EN.

[13] Kriticos D.J., Kean J.M., Phillips C.B., Senay S.D., Acosta H., Haye T. (2017). The Potential Global Distribution of the Brown Marmorated Stink Bug, *Halyomorpha halys,* a Critical Threat to Plant Biosecurity. J. Pest Sci..

[14] Haye T., Gariepy T., Hoelmer K., Rossi J.P., Streito J.C., Tassus X., Desneux N. (2015). Range expansion of the invasive brown marmorated stinkbug, *Halyomorpha halys*: An increasing threat to field, fruit and vegetable crops worldwide. J. Pest Sci..

[15] Damos P., Soulopoulou P., Thomidis T. (2020). First record and current status of the brown marmorated sting bug *Halyomorpha halys* damaging peaches and olives in northern Greece. J. Plant Prot. Res..

[16] Andreadis S.S., Gogolashvili N.E., Fifis G.T., Navrozidis E.I., Thomidis T. (2021). First report of native parasitoids of *Halyomorpha halys* (Hemiptera: Pentatomidae) in Greece. Insects.

[17] Zapponi L., Morten M., Chiesa S.G., Angeli G., Borri G., Mazzoni V., Sofia M., Anfora G. (2022). Brown marmorated stink bug (*Halyomorpha halys*) feeding damage determines early drop in olive crops. J. Appl. Entomol..

[18] Daher E., Cinosi N., Chierici E., Rondoni G., Famiani F., Conti E. (2022). Field and Laboratory Efficacy of Low-Impact Commercial Products in Preventing Olive Fruit Fly, *Bactrocera oleae*, Infestation. Insects.

[19] Daher E., Rondoni G., Cinosi N., Conti E., Famiani F. (2023). Particle Films Combined with Propolis Have Positive Effects in Reducing *Bactrocera oleae* Attacks on Olive Fruits. Horticulturae.

[20] Leskey T.C., Lee D.H., Short B.D., Wright S.E. (2012). Impact of insecticides on the invasive *Halyomorpha halys* (Hemiptera: Pentatomidae): Analysis of insecticide lethality. J. Econ. Entomol..

[21] Kuhar T.P., Kamminga K. (2017). Review of the chemical control research on *Halyomorpha halys* in the USA. J. Pest Sci..

[22] Cianferoni F., Graziani F., Dioli P., Ceccolini F. (2018). Review of the occurrence of *Halyomorpha halys* (Hemiptera: Heteroptera: Pentatomidae) in Italy, with an update of its European and World distribution. Biologia.

[23] Rondoni G., Chierici E., Marchetti E., Nasi S., Ferrari R., Conti E. (2022). Improved Captures of the Invasive Brown Marmorated Stink Bug, *Halyomorpha halys*, Using a Novel Multimodal Trap. Insects.

[24] Peiffer M., Felton G.W. (2014). Insights into the saliva of the brown marmorated stink bug *Halyomorpha halys* (Hemiptera: Pentatomidae). PLoS ONE.

[25] Miles P.W. (1972). The Saliva of Hemiptera. Adv. Insect Phys..

[26] Nielsen A.L., Hamilton G.C. (2009). Seasonal occurrence and impact of *Halyomorpha halys* (Hemiptera: Pentatomidae) in tree fruit. J. Econ. Entomol..

[27] Chen J.H., Avila G.A., Zhang F., Guo L.F., Sandanayaka M., Mi Q.Q., Shi S.S., Zhang J.P. (2020). Field cage assessment of feeding damage by *Halyomorpha halys* on kiwifruit orchards in China. J. Pest Sci..

[28] Schumm Z.R., Alston D.G., Spears L.R., Manlove K. (2020). Impact of Brown Marmorated Stink Bug (Hemiptera: Pentatomidae) Feeding on Tart Cherry (Rosales: Rosaceae) Quality and Yield in Utah. J. Econ. Entomol..

[29] Acebes-Doria A.L., Leskey T.C., Bergh J.C. (2016). Injury to apples and peaches at harvest from feeding by *Halyomorpha halys* (Stål) (Hemiptera: Pentatomidae) nymphs early and late in the season. Crop Prot..

[30] Gömez-Caravaca A.M., Cerretani L., Bendini A., Segura-Carretero A., Fernández-Gutiérrez A., Del Carlo M., Compagnone D., Cichelli A. (2008). Effects of fly attack (*Bactrocera oleae*) on the phenolic profile and selected chemical parameters of olive oil. J. Agric. Food Chem..

[31] Mertoǵlu G., Kumrala N.A. (2018). Economic evaluation of different insecticide applications for control of the olive moth, *Prays oleae* (Bern.), in “Gemlik” olive trees. Acta Hortic..

[32] Giovannini L., Sabbatini-Peverieri G., Marianelli L., Rondoni G., Conti E., Roversi P.F. (2022). Physiological host range of *Trissolcus mitsukurii*, a candidate biological control agent of *Halyomorpha halys* in Europe. J. Pest Sci..

[33] Rondoni G., Chierici E., Giovannini L., Sabbatini-Peverieri G., Roversi P.F., Conti E. (2022). Olfactory responses of *Trissolcus mitsukurii* to plants attacked by target and non-target stink bugs suggest low risk for biological control. Sci. Rep..

[34] Cirilli M., Caruso G., Gennai C., Urbani S., Frioni E., Ruzzi M., Servili M., Gucci R., Poerio E., Muleo R. (2017). The role of polyphenoloxidase, peroxidase, and β-glucosidase in phenolics accumulation in *Olea europaea* L. Fruits under different water regimes. Front. Plant Sci..

[35] Rondoni G., Fenjan S., Bertoldi V., Ielo F., Djelouah K., Moretti C., Buonaurio R., Ricci C., Conti E. (2018). Molecular detection of field predation among larvae of two ladybird beetles is partially predicted from laboratory experiments. Sci. Rep..

[36] Valentin R.E., Maslo B., Lockwood J.L., Pote J., Fonseca D.M. (2016). Real-time PCR assay to detect brown marmorated stink bug, *Halyomorpha halys* (Stål), in environmental DNA. Pest Manag. Sci..

[37] Cribari-Neto F., Zeileis A. (2010). Beta Regression in R. J. Stat. Softw..

[38] Lenth R. (2022). Emmeans: Estimated Marginal Means, Aka Least-Squares Means, R Package Version 1.8.2. https://CRAN.R-project.org/package=emmeans.

[39] Rapoport H.F., Pérez-López D., Hammami S.B.M., Agüera J., Moriana A. (2013). Fruit pit hardening: Physical measurement during olive fruit growth. Ann. Appl. Biol..

[40] Futch S.H., Childers C.C., Mccoy C.W. (2002). Identification of Insect Pests: HS-893/HS142, 11/2002. EDIS.

[41] Spooner-Hart R., Tesoriero L., Hall B.H. (2007). Field Guide to Olive Pests, Diseases and Disorders in Australia.

[42] Rice K.B., Bergh C.J., Bergmann E.J., Biddinger D.J., Dieckhoff C., Dively G., Fraser H., Gariepy T., Hamilton G., Haye T. (2014). Biology, ecology, and management of brown marmorated stink bug (Hemiptera: Pentatomidae). J. Integr. Pest Manag..

[43] Farinelli D., Tombesi S. (2015). Performance and oil quality of “Arbequina” and four Italian olive cultivars under super high-density hedgerow planting system cultivated in central Italy. Sci. Hortic..

[44] Wiman N.G., Parker J.E., Rodriguez-Saona C., Walton V.M. (2015). Characterizing Damage of Brown Marmorated Stink Bug (Hemiptera: Pentatomidae) in Blueberries. J. Econ. Entomol..

[45] Dar S.A., Rather B.A., Wani A.R., Ganie M.A. (2017). Resistance against insect pests by plant phenolics and their derivative dompounds. Chem. Sci. Rev. Lett..

[46] Lattanzio V., Lattanzio V.M.T., Cardinali A. (2015). Role of phenolics in the resistance mechanisms of plants against fungal pathogens and insects. Phytochemistry.

[47] Rondoni G., Bertoldi V., Malek R., Djelouah K., Moretti C., Buonaurio R., Conti E. (2018). *Vicia faba* plants respond to oviposition by invasive *Halyomorpha halys* activating direct defences against offspring. J. Pest Sci..

[48] Serteyn L., Quaghebeur C., Ongena M., Cabrera N., Barrera A., Molina-Montenegro M.A., Francis F., Ramírez C.C. (2020). Induced systemic resistance by a plant growth-promoting rhizobacterium impacts development and feeding behavior of aphids. Insects.

[49] Peterson H.M., Ray S., Ali J.G., Krawczyk G. (2022). Feeding and oviposition by the brown marmorated stink bug, *Halyomorpha halys* (Stål) induce direct and systemic changes in volatile compound emissions from potted peach and tree of heaven. Arthropod-Plant Interact..

[50] Chierici E., Sabbatini-Peverieri G., Roversi P.F., Rondoni G., Conti E. (2023). Phenotypic plasticity in an egg parasitoid affects olfactory response to odors from the plant–host complex. Front. Ecol. Evol..

[51] Suzuki N., Rivero R.M., Shulaev V., Blumwald E., Mittler R. (2014). Abiotic and biotic stress combinations. New Phytol..

[52] Ivancic T., Grohar M.C., Jakopic J., Veberic R., Hudina M. (2022). Effect of Brown Marmorated Stink Bug (*Halyomorpha Halys* Stål.) Infestation on the Phenolic Response and Quality of Olive Fruits (*Olea europaea* L.). Agronomy.

[53] Amiot M.J., Fleuriet A., Macheix J.J. (1986). Importance and Evolution of Phenolic Compounds in Olive during Growth and Maturation. J. Agric. Food Chem..

[54] Boskou D., Blekas G., Tsimidou M. (2005). Phenolic compounds in olive oil and olives. Curr. Top. Nutraceutical Res..

[55] Malheiro R., Casal S., Baptista P., Pereira J.A. (2015). A review of *Bactrocera oleae* (Rossi) impact in olive products: From the tree to the table. Trends Food Sci. Technol..

[56] Fernández-Poyatos M.D.P., Llorent-Martínez E.J., Ruiz-Medina A. (2021). Effect of ripening on the phenolic composition and mineral content of three varieties of olive fruits. Foods.

[57] Seçmeler Ö., Galanakis C.M. (2019). Chapter 8—Olive Fruit and Olive Oil. Innovations in Traditional Foods.

[58] Servili M., Selvaggini R., Esposto S., Taticchi A., Montedoro G.F., Morozzi G. (2004). Health and Sensory Properties of Virgin Olive Oil Hydrophilic Phenols: Agronomic and Technological Aspects of Production That Affect Their Occurrence in the Oil. J. Chromatogr..

[59] Soler-Rivas C., Espı J.C., Wichers H.J. (2000). Review Oleuropein and Related Compounds. J. Sci. Food Agric..

[60] Whitehill J.G.A., Rigsby C., Cipollini D., Herms D.A., Bonello P. (2014). Decreased emergence of emerald ash borer from ash treated with methyl jasmonate is associated with induction of general defense traits and the toxic phenolic compound Verbascoside. Oecologia.

[61] Muñoz E., Lamilla C., Marin J.C., Alarcon J., Cespedes C.L. (2013). Antifeedant, insect growth regulatory and insecticidal effects of *Calceolaria talcana* (Calceolariaceae) on *Drosophila melanogaster* and *Spodoptera frugiperda*. Ind. Crops Prod..

[62] Servili M., Montedoro G. (2002). Contribution of phenolic compounds to virgin olive oil quality. Eur. J. Lipid Sci. Technol..

