# Supplementary material for: Characterization of Olive Fruit Damage Induced by Invasive Halyomorpha halys
Source: Insects. 2023 Oct 31;14(11):848. doi: 10.3390/insects14110848 (PMC10671571; doi:10.3390/insects14110848)
Supplement: Supplementary file 1 [file insects-14-00848-s001.zip › insects-2670092-supplementary.pdf]

Table S1. Phenolic composition (mg/g of fresh weight) of healthy and damaged olive fruits of cultivars Casaliva and Leccino (results are the mean of two independent extractions, each analyzed in duplicate). Means within a row followed by different letters are significantly different for P ≤ 0.05 according to linear model and multiple comparisons procedure.

Cultivar	Compound	Healthy	Damaged	Highly damaged
Casaliva	3,4-DHPEA	0.17 ± 0.016	0.17 ± 0.023	0.17 ± 0.016
	Idrossitirosolo glucoside (T.I.)	n.d.	n.d.	n.d.
	p-HPEA	0.09 ± 0.006	0.1 ± 0.026	0.09 ± 0.037
	Demethyloleuropein	0.47 ± 0.206	2.14 ± 1.179	3.45 ± 0.127
	Verbascoside	0.35 ± 0.109 b	0.28 ± 0.002 b	4.1 ± 0.534 a
	3,4-DHPEA-EDA	0.73 ± 0.04	0.53 ± 0.051	0.65 ± 0.005
	Oleuropein	12.57 ± 0.012 a	9.54 ± 0.69 b	8.13 ± 0.414 b
	P-HPEA-EDA	n.d.	n.d.	n.d.
	Ligustroside	0.26 ± 0.077	0.24 ± 0.028	0.32 ± 0.019
	Rutin	0.2 ± 0.092	0.21 ± 0.03	0.32 ± 0.001
	(+)-1-Acetoxypinoresinol	n.d.	n.d.	n.d.
	(+)-Pinoresinol	n.d.	n.d.	n.d.
	Ligustroside aglicone	n.d.	n.d.	n.d.
	Comselogoside (T.I.)	n.d.	n.d.	n.d.
	**Sum of the phenolic fractions**	14.84 ± 0.534 ab	13.22 ± 0.486 b	17.24 ± 0.897 a
Leccino	3,4-DHPEA	0.23 ± 0.003	0.23 ± 0.034	0.22 ± 0.033
	Idrossitirosolo glucoside (T.I.)	0.22 ± 0.003	0.25 ± 0.037	0.28 ± 0.041
	p-HPEA	0.14 ± 0.002	0.16 ± 0.023	0.14 ± 0.022
	Demethyloleuropein	7.24 ± 0.584 ab	8.97 ± 0.154 a	5.27 ± 0.144 b
	Verbascoside	0.35 ± 0.004	0.64 ± 0.041	0.81 ± 0.012
	3,4-DHPEA-EDA	0.64 ± 0.008 a	0.64 ± 0.011 a	0.58 ± 0.01 b
	Oleuropein	9.12 ± 0.184	6.25 ± 0.387	8.96 ± 0.906
	P-HPEA-EDA	n.d.	n.d.	n.d.
	Ligustroside	0.43 ± 0.005 a	0.29 ± 0.005 c	0.33 ± 0.006 b
	Rutin	0.43 ± 0.005 a	0.23 ± 0.004 c	0.37 ± 0.006 b
	(+)-1-Acetoxypinoresinol	n.d.	n.d.	n.d.
	(+)-Pinoresinol	n.d.	n.d.	n.d.
	Ligustroside aglicone	n.d.	n.d.	n.d.
	Comselogoside (T.I.)	0.08 ± 0.001 a	0.06 ± 0.001 b	0.04 ± 0.005 b
	**Sum of the phenolic fractions**	18.67 ± 0.371 c	17.41 ± 0.659 b	16.73 ± 0.714 a
